# A novel approach to calculating the kinetically derived maximum dose

**DOI:** 10.1007/s00204-022-03229-x

**Published:** 2022-02-01

**Authors:** Lyle D. Burgoon, Claudio Fuentes, Christopher J. Borgert

**Affiliations:** 1 Raptor Pharm & Tox, Ltd., Apex, NC USA; 2 Oregon State University, Corvallis, OR USA; 3 Applied Pharmacology and Toxicology, Inc., Gainesville, FL USA

**Keywords:** Kinetically derived maximal dose, Maximum tolerated dose, KMD, MTD, Toxicokinetics, Michaelis–Menten

## Abstract

The kinetically derived maximal dose (KMD) provides a toxicologically relevant upper range for the determination of chemical safety. Here, we describe a new way of calculating the KMD that is based on sound Bayesian, theoretical, biochemical, and toxicokinetic principles, that avoids the problems of relying upon the area under the curve (AUC) approach that has often been used. Our new, mathematically rigorous approach is based on converting toxicokinetic data to the overall, or system-wide, Michaelis–Menten curve (which is the slope function for the toxicokinetic data) using Bayesian methods and using the “kneedle” algorithm to find the “knee” or “elbow”—the point at which there is diminishing returns in the velocity of the Michaelis–Menten curve (or acceleration of the toxicokinetic curve). Our work fundamentally reshapes the KMD methodology, placing it within the well-established Michaelis–Menten theoretical framework by defining the KMD as the point where the kinetic rate approximates the Michaelis–Menten asymptote at higher concentrations. By putting the KMD within the Michaelis–Menten framework, we leverage existing biochemical and pharmacological concepts such as “saturation” to establish the region where the KMD is likely to exist. The advantage of defining KMD as a region, rather than as an inflection point along the curve, is that a region reflects uncertainty and clarifies that there is no single point where the curve is expected to “break;” rather, there is a region where the curve begins to taper off as it approaches the asymptote (*V*_max_ in the Michaelis–Menten equation).

## Introduction

Regulatory toxicology studies are conducted to help society avoid hazards and unacceptable risks that might be posed by exposure to chemicals. Since toxicity, and thus, hazards, are not intrinsic to the chemical itself but also depend upon the dose and the conditions under which consumers, workers, plants, and animals encounter chemicals (McCarty et al. 2020), avoiding hazards and unacceptable risks requires identifying doses of chemicals that produce no adverse effects; see explanation in our companion paper, Borgert et al. (2021). The goal is to ensure safety by providing regulatory agencies with information about the safe dose range, so they can set safe exposure levels.

Contrast this with investigational toxicology, which is focused on understanding why a chemical is toxic—what is the mode of action, what are the biomarkers that can be used to infer toxicity, are there specific chemical structures that should be avoided in the future?

The difference between regulatory toxicology and investigational toxicology is clear—it is the purpose. The purpose of the studies, the information that they will yield, and how that information will inform decisions is what underlies the differences.

This paper focuses on regulatory toxicology—specifically, how should studies be designed to identify the safe exposure levels? We argue that the old standard, the maximum tolerated dose (MTD), should be abandoned, and that regulatory toxicology studies should embrace a more objectively defined kinetically derived maximum dose (KMD) that we introduce here.

Therefore, what is wrong with MTD studies? Some have argued that toxicity studies at low doses tend to miss toxicity, and that very-high-dose studies are thus warranted (McConnell 1989; Heringa et al. 2020; Woutersen et al. 2020; Slob et al. 2020). They contend that lower doses are inadequate, because lower doses will produce smaller effect sizes, leading to a lower chance of being statistically significant, and thus, increasing the false negative rate. Their solution is to call for studies at higher doses which they believe will increase the statistical power.

Such interpretations of statistical power are misleading. In addition, studies with small sample sizes and lower statistical power actually lead to an increase in false positives (Christley 2010; Gelman and Carlin 2014). In other words, low-power studies done at lower doses where the effect sizes are smaller are more likely to be found significantly different, when they should not be. This is a major contributing factor to the reproducibility crisis we face in toxicology today. These flawed arguments are predicated on the false notion that there must be toxicological effects at all doses, including at lower doses that may truly lack effects.

In addition to the conceptual errors involved in advocating use of the MTD for regulatory toxicology studies, the MTD is rarely relevant to human exposures, may confound identification of the non-hazardous dose range in a variety of ways, and is unnecessary for establishing safety, as we have recently explained (Borgert et al. 2021). Since animals used in an MTD-based study are experiencing unnecessary harm without the counterbalance of the benefits for ensuring human safety, the use of animals in an MTD study is unethical.

Fortunately, better approaches are available. For regulatory purposes, dose-setting based on kinetics is superior to MTD-based dose-setting, and as we have recently described (Borgert et al. 2021).

Setting doses based on the KMD helps to ensure that the study is conducted under conditions of consistent toxicokinetics and that any adverse effects observed are due to dose-relevant mode(s) of action. This is accomplished by ensuring that the doses employed in the study are constrained to the same side of the toxicokinetic conditions—either the study is conducted below saturation of elimination kinetics, or at the point of saturation of elimination kinetics and above. Crossing that boundary, where the enzymes become saturated, triggers different modes of action, and thus, including both dose ranges within the same study requires a clear delineation of interpretation between the two dose ranges, a delineation typically ignored in regulatory interpretations.

Here, we introduce a new, mathematically rigorous way to identify the region where the KMD exists. We believe that a new KMD approach is necessary as the existing method requires assumptions that can be problematic. Specifically, the existing KMD approach requires the use of the area under the toxicokinetic curve (AUC) at 24 h (Saghir 2015; Saghir et al. 2012). The assumption when using the AUC in this way is that the overall shapes of the various possible blood concentration curves will be congruent. However, it is well known that multiple, very different, blood concentration curves can all yield the same AUC. It is also well established statistically that using a small number of replicates increases the likelihood that the sample distribution of AUCs and the sample distribution of the elimination curves will differ markedly from the population distribution of AUCs and elimination curves. In other words, a small number of replicates are far less likely to replicate the population than one would hope; meaning that any estimate of where the KMD region might lie is more likely to be biased (as in deviating from the population distribution) than not. Compounding these complications is the fact that toxicity is often the result of peak plasma concentrations rather than the total dose (which the AUC represents), as is the case for chloroform and perchloroethylene (reviewed in Borgert et al. 2015); hence, there is no compelling reason to ground the relationship between administered dose and blood level on the AUC rather than on a different kinetic parameter.

Finally, we find that the use of a point estimate for the KMD is not mathematically rigorous. The reason is simple—there is not one single point where the rate of the elimination curve moves from being linear to nonlinear. Said another way—our eyes may perceive that the linear aspect of the elimination curve is linear, but in reality, the slope, or rate (or first derivative) of the elimination curve is constantly changing as a function of time (or concentration when examining the Michaelis–Menten curve). While a portion of the rate curve may appear to be linear, it is quasi-linear at best (where quasi-linear means having an appearance that is linear, meaning no change in the slope function, when in fact there are small changes in the slope function). Due to various biological processes, the rate of the elimination kinetics curve is actually described by a system-wide Michaelis–Menten equation. Thus, mathematically, we believe that it is better to refer to the KMD as a region, representing uncertainty, where the rate curve begins to demonstrably approach the asymptote, where the asymptote represents system-wide saturation of the elimination mechanism.

We were motivated, for these reasons, to develop a new KMD approach that does not rely on the same set of problematic assumptions, but instead is closely grounded in established biochemical and pharmacological theory. Our approach uses toxicokinetic data to estimate the Michaelis–Menten mechanics that undergird the toxicokinetics. Specifically, Michaelis–Menten mechanics represent the slope of the toxicokinetic data—it is actually Michaelis–Menten mechanics that determine the KMD, as the saturation of enzyme kinetics is represented as the Michaelis–Menten curve approaching the high concentration asymptote. Using toxicokinetic data to estimate the Michaelis–Menten parameters, we can do a better job of estimating the region where the KMD exists using mathematical algorithms designed to identify maximal curvature, such as the “kneedle” algorithm (Satopaa et al. 2011) which is used to find “knees” or “elbows” in continuous, scale-free curves.

## Defining the KMD using the Michaelis–Menten equation

When a drug or chemical (we will use the term “chemical” broadly throughout to also include drugs) is ingested, or somehow introduced into the circulation, the absorption, distribution, metabolism, and excretion (ADME) of the chemical is governed by two general phenomena, first, by physical–chemical properties of the chemical and the system, that is, adsorption and partitioning between lipid and aqueous compartments, and second, by the action of specific proteins. With respect to the latter—active proteins such as transporters, enzymes, etc.—a chemical will typically bind or interact with in a way that causes a biologically important alteration. For instance, chemicals may bind plasma proteins, facilitating their transport throughout the body and/or hinder their metabolism and excretion. Other proteins may facilitate the efflux of chemicals or their metabolites into urine, bile ducts, or into the intestinal lumen. Some proteins may enzymatically transform the chemical into a metabolite, or conjugate the chemical or metabolite to facilitate excretion.

Chemical and protein interactions are governed by Michaelis–Menten mechanics. Michaelis–Menten mechanics provide a mathematical way of understanding the velocity of product formation as a function of the substrate concentration. For instance, the rate of production of a chemical metabolite by an enzyme is governed by the Michaelis–Menten equation

$$v=\frac{{V}_{\mathrm{max}}[S]}{{K}_{m}+[S]},$$

where *v* is the reaction rate, *V*_*max*_ is the maximum reaction rate, *K*_*m*_ is the substrate concentration that results in 50% of *V*_*max*_, and [*S*] is the substrate concentration.

Michaelis–Menten kinetics governs all processes where a chemical interacts with a specific active protein. Chemical binding to plasma binding proteins is governed by Michaelis–Menten kinetics, where the product is the chemical-bound protein complex. The same is true for chemical/ligand binding to receptors. In the case of transporter proteins, such as the multidrug resistance 2 (MRP2) protein, the product is the effluxed chemical.

If we consider ADME as a system, it is ultimately driven by numerous chemical–protein interactions. As each chemical–protein interaction is governed by their own Michaelis–Menten equation, the function of the system as a whole is the result of a system of Michaelis–Menten equations, where some equations govern the appearance of the chemical in the system (e.g., absorption and reabsorption), others govern the distribution of the chemical into and out of organ compartments (e.g., distribution), while others govern the disappearance of the chemical (e.g., metabolism and excretion). The rates of absorption/reabsorption, distribution, metabolism, and excretion are all the result of the sums of the Michaelis–Menten equations for the proteins (including enzymes and channels) and other macromolecules that govern different parts of the ADME process. We know that this is true given the fact that physiologically based pharmacokinetic models work, and this is no more than an extension of the concept of a system of continuous stirred tank reactor systems. The sums of the Michaelis–Menten equations across all of ADME can be brought together into a larger system-level Michaelis–Menten equation that governs the rate of the observed pharmacokinetic curve.

Thus, the observed pharmacokinetic curve has a Michaelis–Menten rate. By definition, that rate is the first derivative of the pharmacokinetic curve. Likewise, as the Michaelis–Menten curve governs the observed pharmacokinetic curve, the observed pharmacokinetic curve also adheres to certain known characteristics of Michaelis–Menten kinetics. Chief among these is the observation that as the substrate/chemical/ligand concentration increases, the velocity asymptotically increases toward the maximum velocity, *V*_*max*_. In enzyme and receptor kinetics, we refer to this phenomenon as enzyme or receptor saturation. The key point being that one does not need to experience 100% occupation of a set of enzymes or receptors in the cell to see saturation. Rather, the key idea is that the increase in the slope (the second derivative of the pharmacokinetic curve) declines as the amount of substrate/chemical/ligand increases, and that there is a point where the increase in the slope slows so much that the system acts or responds as if the system is saturated.

## Finding the KMD

Our approach to finding the KMD is different from others. Although we use toxicokinetic data, our approach is based on Michaelis–Menten mechanics, and then, a mathematical analysis called the “kneedle” algorithm is used to identify the point of “diminishing returns”—the point at which the change in slope clearly demarcates the curve being nearly indistinguishable from the asymptote (Satopaa et al. 2011). The region within which that point lies, also known as a knee or elbow in the curve, is the KMD.

Our approach starts by reverse engineering the system-wide Michaelis–Menten mechanics that result in the toxicokinetic curve. Our view is that elimination kinetics is governed by a system of Michaelis–Menten functions, as proteins (and mostly enzymes) are driving ADME. Each enzyme has its own Michaelis–Menten equation that govern it, but the sum of all of these working together as a system results in a system-wide, overall Michaelis–Menten function. It is this overall, system-wide function that governs the observed toxicokinetic curve.

Here, we use a Bayesian framework to estimate *V*_*max*_ and *K*_*m*_ using the toxicokinetic data. This approach applies Bayes Theorem to obtain a final (posterior) distribution of plausible *V*_*max*_ and *K*_*m*_ values by adjusting observed data based on prior knowledge. We used information from previously published, peer-reviewed studies and government reports to build our prior distribution. Applying Bayesian analysis with differential equations, we can use the toxicokinetic data to estimate likely values for *V*_*max*_ and *K*_*m*_. The resulting distributions of likely *V*_*max*_ and *K*_*m*_ values can then be used to generate a set of Michaelis–Menten equations that are likely to represent the slope function for the toxicokinetic data, and thus estimate a range of KMD values. A similar analysis could be conducted by classical/frequentist statistical approaches using maximum-likelihood approaches rather than prior knowledge. Estimates for *V*_*max*_ and *K*_*m*_ can be used to build the Michaelis–Menten curve by applying the Michaelis–Menten equation.

In this specific example, our calculations are based on the following:

$$y \sim Normal\left(u,\sigma \right)$$

$$\sigma \sim HalfCauchy\left(1\right)$$

$${V}_{max} \sim Normal[25, 2;\left(0,infinity\right)]$$

$${K}_{m} \sim Normal[\mathrm{11,2};\left(0, infinity\right)]$$

$$\mu \sim {V}_{max}\frac{[S]}{{K}_{m}+[S]},$$

where σ is the standard deviation; µ is the instantaneous slope of the toxicokinetic curve, expressed as the Michaelis–Menten equation. *V*_*max*_ and *K*_*m*_ have truncated normal distributions, with hyperparameters derived from the literature. Less informative priors can also be used for *V*_*max*_ and *K*_*m*_ when prior information is lacking. What we are doing is calculating the distribution of likely values of *V*_*max*_ and *K*_*m*_ using the prior distributions (prior or existing information from the literature to set the prior distributions), and informing these priors using the toxicokinetic data. Thus, we are estimating *V*_*max*_ and *K*_*m*_ using the toxicokinetic data as input to an ordinary differential equation (ODE) solver and specifically minimizing the difference between the observed toxicokinetic data and the simulated value from the ODE solver.

**Fig. 1:**
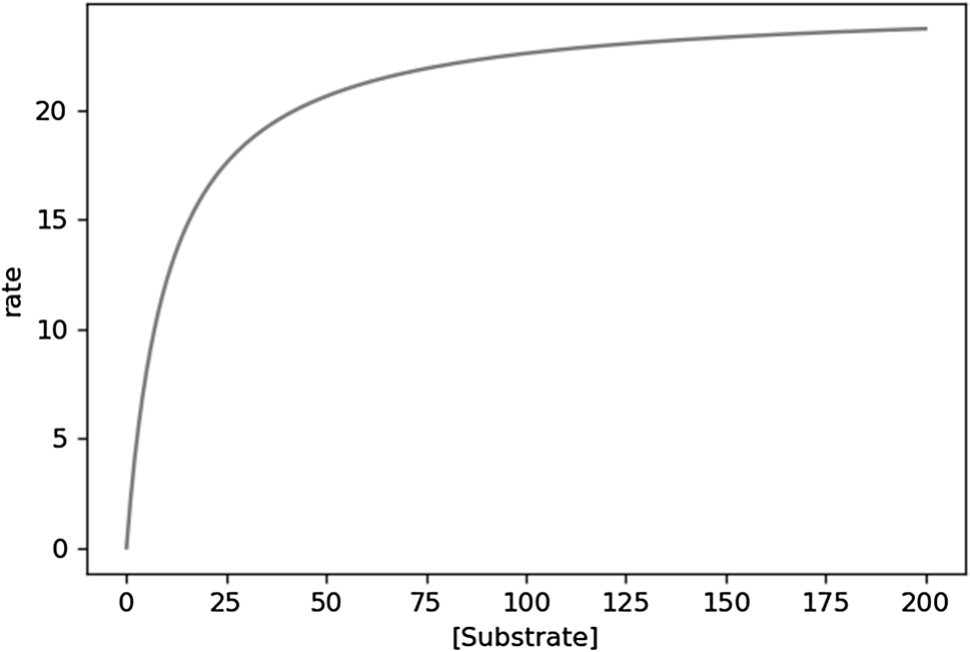
An example of Michaelis–Menten curve. The *x*-axis is the substrate concentration, and the y-axis is the enzyme conversion rate for the substrate. As the substrate concentration increases, the rate increases. This behavior is asymptotic, such that the slope of the rate decreases with increasing substrate concentration—as the asymptote is reached

Figure 1 presents the single Michaelis–Menten curve built from the mean of the *V*_*max*_ and *K*_*m*_ distributions.

To calculate the KMD we used the kneedle algorithm. This algorithm finds the point on a curve where the slope approaches an asymptote (i.e., the flat part of the curve at higher concentrations). This is also known as the point of “diminishing returns” or the “knee” (Satopaa et al. 2011). The challenge to identifying this point on an exponential curve, or something similar such as the Michaelis–Menten curve, is that the point is not scale-invariant, meaning that the highest substrate concentration and rate plotted will alter the point at which the kneedle algorithm identifies the “knee”. In our experience, looking for the kneedle with the highest plotted concentration corresponding to 90% or 95% of *V*_max_ tends to be sufficiently close to the asymptote.

**Fig. 2:**
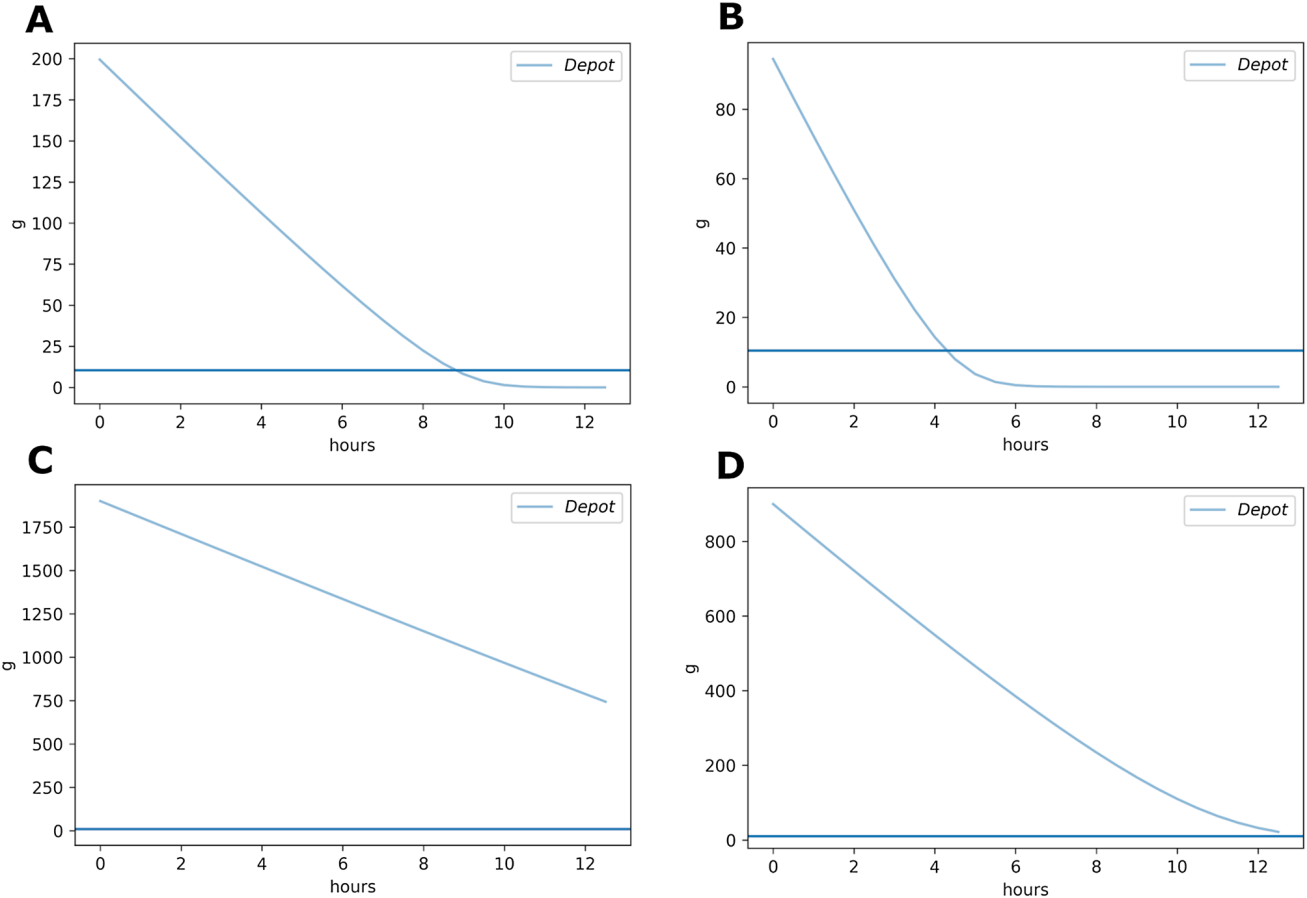
Elimination kinetics curves. **A** The elimination kinetics curve for an enzyme or enzyme system with a *V*_max_ = 25 and a *K*_m_ = 10.5 and a starting concentration equivalent to the rate at 95% of the *V*_max_. **B** The elimination kinetics curve for an enzyme or enzyme system with a *V*_max_ = 25 and a *K*_m_ = 10.5 and a starting concentration equivalent to the rate at 90% of the *V*_max_. **C** The elimination kinetics curve for an enzyme or enzyme system with a *V*_max_ = 100 and a *K*_m_ = 100 and a starting concentration equivalent to the rate at 95% of the *V*_max_. **D** The elimination kinetics curve for an enzyme or enzyme system with a *V*_max_ = 100 and a *K*_m_ = 100 and a starting concentration equivalent to the rate at 90% of the *V*_max_

The reason for suggesting 90% or 95% is simple: the goal is to choose a point that is near the concentration at which the enzyme behaves as if it were saturated. Saturable behavior is noted as being very near to zero-order elimination kinetics, i.e., that the elimination kinetics are linear. Keep in mind that the elimination kinetics are the same as the slope of the curve. As the slope will never be linear for the Michaelis–Menten curve, we instead look for the region where the change is slope is the smallest—that is, the asymptotic region. Based on our experiences, the region from 90 to 95% of the *V*_*max*_ is quite suitable (see Table 1 and Fig. 2).

**Table 1:** Slopes over time for different enzymatic parameters (see Fig. 2)

Time range (h)	*V*_max_: 25 *K*_m_: 10.5 95% *V*_max_ Slope	*V*_max_: 25 *K*_m_: 10.5 90% *V*_max_ Slope	*V*_max_: 100 *K*_m_: 100 95% *V*_max_ Slope	*V*_max_: 100 *K*_m_: 100 90% *V*_max_ Slope
0.0–0.5	− 23.71	− 22.36	− 94.94	− 89.77
0.5–1.0	− 23.63	− 22.02	− 94.82	− 89.28
1.0–1.5	− 23.54	− 21.60	− 94.68	− 88.74
1.5–2.0	− 23.44	− 21.06	− 94.55	− 88.15
2.0–2.5	− 23.33	− 20.33	− 94.40	− 87.50
2.5–3.0	− 23.19	− 19.33	− 94.25	− 86.78
3.0–3.5	− 23.04	− 17.89	− 94.09	− 85.98
3.5–4.0	− 22.85	− 15.78	− 93.92	− 85.08
4.0–4.5	− 22.63	− 12.70	− 93.74	− 84.08
4.5–5.0	− 22.36	− 8.63	− 93.56	− 82.94
5.0–5.5	− 22.03	− 4.55	− 93.36	− 81.66
5.5–6.0	− 21.62	− 1.84	− 93.14	− 80.18
6.0–6.5	− 21.07	− 0.63	− 92.92	− 78.49
6.5–7.0	− 20.35	− 0.20	− 92.68	− 76.54
7.0–7.5	− 19.36	− 0.06	− 92.42	− 74.26
7.5–8.0	− 17.94	− 0.02	− 92.15	− 71.59
8.0–8.5	− 15.85	− 0.01	− 91.85	− 68.45
8.5–9.0	− 12.80	0.00	− 91.53	− 64.74
9.0–9.5	− 8.75	0.00	− 91.19	− 60.37
9.5–10.0	− 4.65	0.00	− 90.83	− 55.24
10.0–10.5	− 1.90	0.00	− 90.43	− 49.30
10.5–11.0	− 0.65	0.00	− 90.00	− 42.62
11.0–11.5	− 0.21	0.00	− 89.53	− 35.40
11.5–12.0	− 0.06	0.00	− 89.01	− 28.06
12.0–12.5	− 0.02	0.00	− 88.45	− 21.12

The substrate concentration at 95% of the *V*_*max*_ can be found algebraically as

$$v=\frac{{V}_{max}\left[S\right]}{{K}_{m}+\left[S\right]}$$

$$\left[S\right]=-\frac{v{K}_{m}}{v-Vmax}$$

$$\left[S\right]=-\frac{\left(0.95{V}_{max}\right){K}_{m}}{0.95{V}_{max}-{V}_{max}}$$

$$\left[S\right]=-\frac{\left(0.95{V}_{max}\right){K}_{m}}{-0.05{V}_{max}}$$

$$\left[S\right]=19{K}_{m}.$$

Similarly, we can find the substrate concentration at 90% of the *V*_max_

$$\left[S\right]=-\frac{\left(0.90{V}_{max}\right){K}_{m}}{0.90{V}_{max}-{V}_{max}}$$

$$\left[S\right]=-\frac{\left(0.90{V}_{max}\right){K}_{m}}{-0.10{V}_{max}}$$

$$\left[S\right]=9{K}_{m}.$$

We calculated the slope of the curves for *V*_*max*_ = 25.0 and *K*_*m*_ = 10.5 and *V*_*max*_ = 100 and *K*_*m*_ = 100 to show the slope of the elimination curves. We used 95% and 90% of the *V*_max_ as our maximum substrate concentrations and the starting concentration. Table 1 shows the slopes of the elimination curves (Fig. 2) for at 95% and 90% of the *V*_max_.

It is clear that the slope is not stable, or truly linear, for large segments of the curve, but one can see that the change in slope is much smaller at the earlier time-points (where the substrate concentration is closer to the asymptote)—this is the range where saturation is clearly more evident (as noted by the smaller change in slope). The starting concentration at 90% of the *V*_*max*_ still shows smaller changes in slope, and is thus still near the saturable kinetics.

### An Application Example

**Fig. 3:**
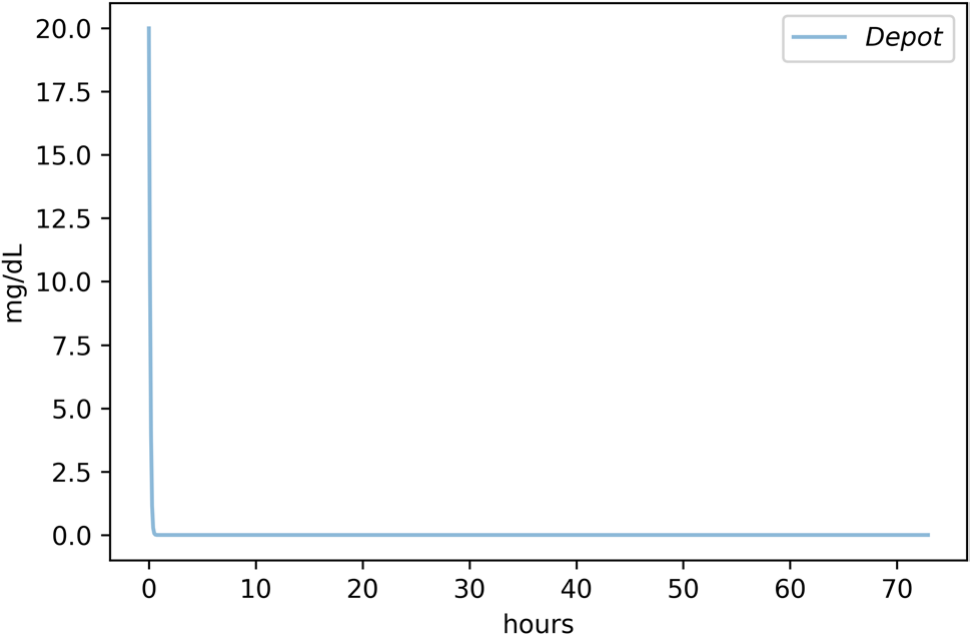
Elimination kinetic curve for alcohol. The rate of alcohol elimination is governed by Michaelis–Menten mechanics. To demonstrate how we use PK data to estimate the *V*_max_ and *K*_m_, we needed to simulate the alcohol elimination curve using empirical estimates of *V*_max_ and *K*_m_. In a typical situation, we would start with the PK data, and then, we would use our Bayesian approach to solve the Michaelis–Menten equation to obtain the *V*_max_ and *K*_m_ values

Typically, we will have toxicokinetic data available for a chemical of interest, and we want to estimate the KMD along with our uncertainty in that estimate. In this instance, we have generated a model set of toxicokinetic data using the known *K*_*m*_ and *V*_*max*_ for liver alcohol dehydrogenase in males and females for ethanol metabolism. For the purposes of this example, we obtained the toxicokinetic data from our toxicokinetics simulator, which uses Michaelis–Menten mechanics to model the system-wide elimination rate. We set the *V*_*max*_ = 175 mg/kg-hr and the *K*_*m*_ = 11.8 mg/dL, which is within the range reported by Baraona et al. (2001). This results in a blood concentration elimination curve shown in Fig. 3.

**Fig. 4:**
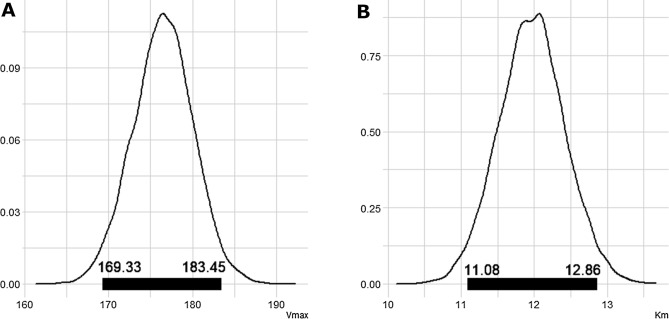
Bayesian estimates of *V*_max_ and *K*_m_ from the Elimination Kinetic Curve for Alcohol. We used a Bayesian approach to estimate the kinetic parameters, *V*_max_ and *K*_m_, associated with the elimination kinetic curve for alcohol. The Michaelis–Menten equation serves as the slope of the pharmacokinetic curve/elimination kinetic curve for alcohol. The histograms show the distribution of the *V*_max_ and *K*_m_ values estimated using the Bayesian approach. The dark lines inside each histogram depict the 95% Bayesian credible interval (the central 95% most likely values for each parameter). We used these values, along with the 90% *V*_max_ and 95% *V*_max_ values to estimate the region where the KMD is most likely to exist

The first step in estimating the KMD is to estimate the system-level *V*_*max*_ and *K*_*m*_. We used a differential equation solver in a Bayesian context to estimate the *V*_*max*_ and *K*_*m*_ values from the Michaelis–Menten equation; however, one could also use a maximum-likelihood approach. The solver gives us a distribution of plausible *V*_*max*_ and *K*_*m*_ values that drive the elimination kinetics. Our Bayesian analysis estimates that *V*_*max*_ has a mean of 176.49 mg/kg-hr, with lower and upper limits of 169.33 mg/kg-hr and 183.45 mg/kg-hr, and that *K*_*m*_ has a mean of 11.97 mg/dL with lower and upper limits of 11.08 mg/dL and 12.86 mg/dL, respectively (Fig. 4). Note that these values for *V*_*max*_ and *K*_*m*_ are similar to the values we used to generate the elimination kinetic curve (*V*_*max*_ = 175 mg/kg-hr and the *K*_*m*_ = 11.8 mg/dL).

**Fig. 5:**
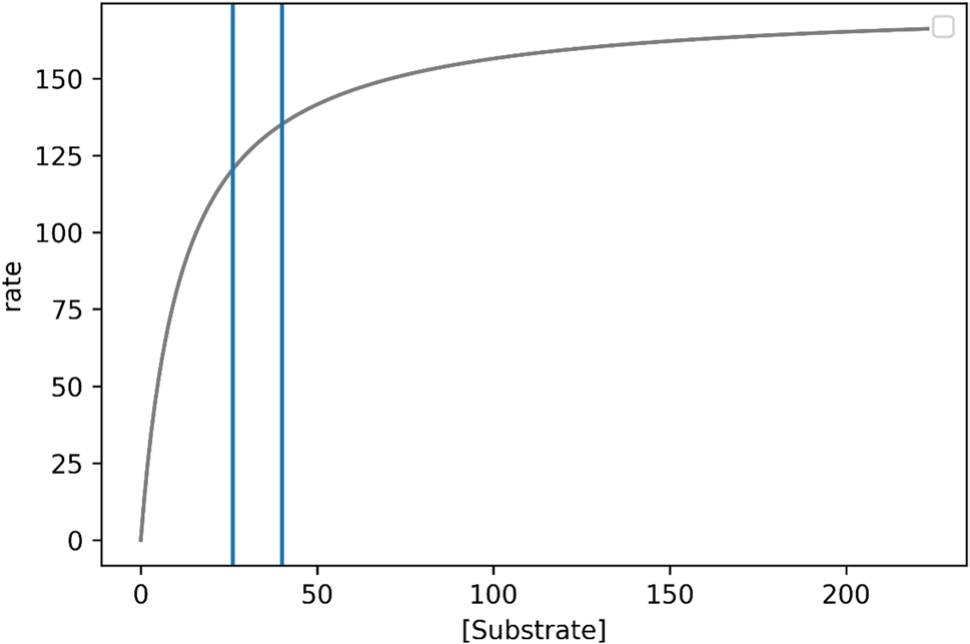
Bounded range of the KMD. We calculated the upper and lower bounds of the KMD for both the 90% *V*_max_ and 95% *V*_max_ values. The substrate concentrations at 90% and 95% *V*_max_ are 106.2 mg/dL and 224.2 mg/dL, respectively. We use the lower bound of *V*_max_, 165 mg/kg-hr, and the lower bound of *K*_m_, 11 mg/dL, with the substrate concentration at 90% of *V*_max_ (106.2 mg/dL) to estimate the lower bound of the range that contains the KMD. We also use upper bounds for *V*_max_ (184 mg/kg-hr) and *K*_m_ (13 mg/dL) and the substrate concentration at 95% *V*_max_ (224.2 mg/dL) to estimate the upper bound of the range that contains the KMD. Thus, the KMD is bound at 25 mg/dL and 43 mg/dL (vertical lines). The mid-point of this range is 34 mg/dL

Next, we input these plausible values and the upper and lower bounds of *V*_max_ and *K*_m_ along with the alcohol concentration at 90% and 95% *V*_max_ into the KMD kneedle algorithm to estimate the range where the KMD is likely. The substrate concentrations at 90% and 95% *V*_max_ are 106.2 mg/dL and 224.2 mg/dL, respectively. We use the lower bound of *V*_max_, 165 mg/kg-hr, and the lower bound of *K*_m_, 11 mg/dL, with the substrate concentration at 90% of *V*_max_ (106.2 mg/dL) to estimate the lower bound of the range that contains the KMD. We also use upper bounds for *V*_max_ (184 mg/kg-hr) and *K*_m_ (13 mg/dL) and the substrate concentration at 95% *V*_max_ (224.2 mg/dL) to estimate the upper bound of the range that contains the KMD. Thus, the KMD is bound at 25 mg/dL and 42 mg/dL (Fig. 5). The mid-point of this range is 34 mg/dL, which we calculated by taking the most plausible values for *V*_max_ and *K*_m_, and using the mean of the 90% *V*_max_ and 95% *V*_max_ as the maximum concentration. Thus, we estimate a plausible range for the KMD to be between 25 and 42 mg/dL with the most likely value being near 34 mg/dL.

## Conclusions

We have demonstrated a mathematically tractable approach based on established principles from biochemistry and pharmacology to identify the range where the KMD likely exists. Thus, we reject the argument that it is not possible to identify the region where a toxicokinetic curve begins to approach enzymatic saturation. Rather, we argue that the notion of enzyme saturation is a well-known and well-recognized concept from the fields of biochemistry and enzymology, governed by the well-established principle of the Michaelis–Menten equation.

We also recognize that there are challenges in identifying the KMD using only the area under the curve (AUC) to represent the pharmacokinetic profile. It is important to note that there is an extensive literature that demonstrates that more than one curve shape can result in the same AUC over any given timeframe. This means that the ADME/kinetics can be substantially different between several curves that all have the same AUC. One of the most obvious examples of this occurs with drugs that have high oral bioavailability, such as ibuprofen (Atkinson et al. 2015; Pavliv et al. 2011). Pavliv et al. (2011) compared the pharmacokinetic profile of ibuprofen administered per os as an 800 mg oral tablet or infused over 5–7 min in 200 mL intravenous saline solution containing 4 mg/mL ibuprofen. Both administrations were equally well tolerated. Figure 1 and Table 1 of their publication show clearly that IV infusion produced a higher Cmax and more rapid tmax, but identical AUC over 12 h compared to oral administration. Since toxicity can depend upon peak plasma concentrations rather than total dose (AUC), as is the case for chloroform and perchloroethylene (reviewed in Borgert et al. 2015), there is no compelling reason to ground the relationship between administered dose and blood level on the AUC rather than on a different kinetic parameter.

Thus, we devised a strategy that refocuses the attention on identifying a KMD where it is mathematically more tenable—the slope function of Michaelis–Menten mechanics. To accomplish this, we describe how a differential equation solver can be used to estimate the Michaelis–Menten parameters, *V*_max_ and *K*_m_, using only the pharmacokinetics data. We then solved the problem of identifying the range where the KMD exists by recasting the problem as a “diminishing returns” problem, where we aim to identify the point on a curve where slope becomes asymptotic. We used the well-established kneedle algorithm to help us identify the KMD region. Consequently, we have demonstrated not only is it possible to estimate a KMD, but that recasting the pharmacokinetics back to basic biochemical and pharmacological principles makes the identification of the region containing the KMD tractable.
